# *Ostα*^*−/−*^ mice are not protected from western diet‐induced weight gain

**DOI:** 10.14814/phy2.12263

**Published:** 2015-01-27

**Authors:** Christine L. Hammond, Sadie G. Wheeler, Nazzareno Ballatori, Patricia M. Hinkle

**Affiliations:** Department of Environmental Medicine, University of Rochester School of Medicine, Rochester, New York, USA; Department of Pharmacology and Physiology, University of Rochester School of Medicine, Rochester, New York, USA

**Keywords:** Bile acids, cholesterol, high‐fat diet, lipid absorption, organic solute transporter

## Abstract

Organic solute transporter*α*‐OST*β* is a bile acid transporter important for bile acid recycling in the enterohepatic circulation. In comparison to wild‐type mice, *Ostα*^*−/−*^ mice have a lower bile acid pool and increased fecal lipids and they are relatively resistant to age‐related weight gain and insulin resistance. These studies tested whether *Ostα*^*−/−*^ mice are also protected from weight gain, lipid changes, and insulin resistance which are normally observed with a western‐style diet high in both fat and cholesterol (WD). Wild‐type and *Ostα*^*−/−*^ mice were fed a WD, a control defined low‐fat diet (LF) or standard laboratory chow (CH). Surprisingly, although the *Ostα*^*−/−*^ mice remained lighter on LF and CH diets, they weighed the same as wild‐type mice after 12 weeks on the WD even though bile acid pool levels remained low and fecal lipid excretion remained elevated. Mice of both genotypes excreted relatively less lipid when switched from CH to LF or WD. WD caused slightly greater changes in expression of genes involved in lipid transport in the small intestines of *Ostα*^*−/−*^ mice than wild‐type, but the largest differences were between CH and defined diets. After WD feeding, *Ostα*^*−/−*^ mice had lower serum cholesterol and hepatic lipids, but *Ostα*^*−/−*^ and wild‐type mice had equivalent levels of muscle lipids and similar responses in glucose and insulin tolerance tests. Taken together, the results show that *Ostα*^*−/−*^ mice are able to adapt to a western‐style diet despite low bile acid levels.

## Abstract (precis)

Mice lacking the organic solute transporter (OST) have abnormally low bile acid pools and are resistant to age‐related weight gain. These experiments tested whether *Ostα*^−/−^ mice are also resistant to western diet‐induced weight gain. Despite low bile acid pools and high fecal lipid excretion, *Ostα*^−/−^ mice gained weight as rapidly as wild‐type mice.

## Introduction

The organic solute and steroid transporter (OST*α*‐OST*β*; SLC51) is an important transporter involved in the enterohepatic recycling of bile acids (Ballatori et al. 2013). Located on the basolateral side of the ileal enterocyte, OST*α*‐OST*β* transports intracellular bile acids into the portal blood. Bile acids play a role in the intestinal absorption of dietary fats and cholesterol, as well as cholesterol homeostasis (Hofmann 2009). Bile acids are synthesized from cholesterol in the liver primarily via the cholesterol 7*α*‐hydroxylase (CYP7A1) pathway, secreted into bile, then stored in the gallbladder and released into the duodenum following a meal. In the intestinal lumen, bile acids facilitate the absorption of dietary cholesterol and fats into enterocytes through emulsification and the formation of micelles. Bile acids are thought to activate signaling pathways through nuclear farnesoid X receptor (FXR) and a membrane G protein‐coupled receptor (TGR5), which are important for the regulation of cholesterol, triglyceride, glucose, and bile acid levels, as well as energy metabolism (Lefebvre et al. 2009; Zhou and Hylemon 2014). Alterations in bile acid transport, synthesis, concentration, and composition can lead to cholestastic liver disease, fatty liver disease, diabetes, and obesity.

Studies in mice deficient in OST*α*‐OST*β* transport have confirmed the important role that OST*α*‐OST*β* has in the enterohepatic circulation through the demonstration of defects in bile acid and sulfated steroid distribution (Ballatori et al. 2008). Although the absorption of bile acids is impaired in the intestines of *Ostα*^*−/−*^ mice, fecal bile acids are not elevated and bile acid levels in liver, bile‐filled gallbladder, and intestine (the bile acid pool) are lower than in wild‐type mice. This was counterintuitive until it was realized that bile acid synthesis is reduced because expression of *Cyp7a1* is decreased in livers of *Ostα*^*−/−*^ mice (Ballatori et al. 2008; Rao et al. 2008). The decrease in *Cyp7a1* has been attributed to an increase in FXR‐mediated FGF15 signaling from the ileum; FGF15 acts in the liver to inhibit *Cyp7a1* (Ballatori et al. 2008; Rao et al. 2008; Lan et al. 2012). Although *Cyp7a1* is decreased in *Ostα*^*−/−*^ mice, serum cholesterol and triglyceride levels remain normal or low (Ballatori et al. 2008; Rao et al. 2008).

As expected from the relationship between bile acids and lipid absorption, fecal lipid and neutral sterol excretion were higher in the *Ostα*^*−/−*^ mice, suggesting a defect in lipid and cholesterol absorption (Rao et al. 2008; Wheeler et al. 2014). Supplementation with cholic acid lowered fecal lipid excretion and restored cholesterol absorption in the *Ostα*^*−/−*^ mice to wild‐type levels (Lan et al. 2012; Wheeler et al. 2014). We also observed that *Ostα*^*−/−*^ mice were thinner than wild‐type mice as they aged and proposed that this resulted in part from a decrease in lipid absorption due to the reduced bile acid pool (Wheeler et al. 2014). *Ostα*^*−/−*^ mice were partially protected from the insulin resistance that develops in wild‐type mice as they age, apparently because the *Ostα*^*−/−*^ mice had decreased muscle lipid accumulation and improved insulin responses in muscle (Wheeler et al. 2014). Additionally, the life expectancy of male *Ostα*^*−/−*^ mice was slightly increased (Wheeler et al. 2014).

Given the importance of bile acids in lipid absorption and the defect in lipid absorption in aging *Ostα*^*−/−*^ mice, we predicted that *Ostα*^*−/−*^ mice would be protected from the weight gain, fat accumulation and insulin resistance that normally occur when mice are placed on a western‐style diet with high fat and cholesterol levels. In contrast to this expectation, we found that *Ostα*^*−/−*^ mice experienced rapid weight gain on a high‐fat diet despite low bile acid pools and high lipid excretion compared with wild‐type mice.

## Materials and Methods

### Animals

*Ostα*^*−/−*^ mice were generated and maintained on a C57Bl/6 background as described (Li et al. 2007; Ballatori et al. 2008). Mice were housed in a full barrier facility at the University of Rochester School of Medicine with a 12 h light/dark cycle and maintained on a standard chow (CH) (Laboratory Autoclavable Rodent Diet 5010; Purina Mills, St Louis, MO). At 2 months of age, male mice were switched to low fat control diet (LF) (D12450B Research Diets, New Brunswick, NJ), and 2 weeks later half were switched to the western diet (WD) (D12079B Research Diets). Percentages of the main dietary components of these diets are presented in Table 1. A second control group of mice remained on CH for the duration of the experiment. Mice were housed 2–3/cage, weighed weekly, and given free access to food and water unless otherwise noted. At the end of the study (wk 12), mice were fasted overnight, anesthetized with pentobarbital sodium (50 mg/kg ip), and tissues and whole blood were collected for analysis. The results described here were obtained using mice that had not been used in any previous studies. All procedures were approved by the University of Rochester Animal Care and Use Committee, according to criteria outlined in the “Guide for the Care and Use of Laboratory Animals” issued by the Institute of Laboratory Animal Research of the National Academy of Sciences.

**Table 1.** Main components of LF, WD, and CH diets. Dietary components supplied by the manufacturers are shown. The major sources of protein are casein in LF and WD and a variety of vegetable proteins in CH. The major sources of fat are soybean oil and lard in LF and CH and milk fat in WD

	LF		WD		CH	
	g%	kcal%	g%	kcal%	g%	kcal%
Protein	19.2	20	20.0	17	24.6	28.7
Carbohydrate	67.3	70	50.0	43	50.1	58.5
Cornstarch	29.9		5.0		38.4	
Maltodextrin 10	3.3		10.0			
Sucrose	33.2		34.1		1.1	
Glucose					0.25	
Fructose					0.28	
Fat	4.3	10	21.0	41	4.5	12.7
Cholesterol	0.005		0.21		0.003	
kcal/g	3.85		4.70		4.14	

### Analysis of food consumption and fecal production

Food in each cage was weighed over a 24 h period. The weight difference was divided by the number of mice per cage to estimate daily food consumption. To estimate fecal production, feces were collected from cages, dried for 72 h in a drying oven and weighed. Weights were divided by the number of mice per cage and time spent in cage to estimate fecal production.

### Analysis of fecal lipids

Fecal lipid content was determined gravimetrically (Newberry et al. 2006) from dried feces. Briefly, 0.5 g of dried feces were homogenized in water or phosphate‐buffered saline and extracted using chloroform/methanol (2:1). A portion of the organic phase was dried in a vacuum evaporator to determine lipid mass.

### Bile acid analysis

The bile acid pool samples included liver, gallbladder, and small intestines of mice after an overnight fast. Bile acids were extracted from dried feces and bile acid pool samples using methanol as described (Setchell et al. 1995) and total bile acids were analyzed using an enzymatic assay (Mashige et al. 1981).

### Liver and muscle lipid analysis

Quadriceps and 100–200 mg liver samples were collected from overnight‐fasted mice, flash frozen in liquid nitrogen, and stored at −80°C until analysis. Frozen tissues were homogenized in 7.5 mL chloroform/methanol (2:1), sonicated for 1 h, and filtered using syringe‐driven 0.45 μm PTFE filter units. Sulfuric acid (2.5 mL of 0.05%) was added to filtered extractions and samples were centrifuged for 2 min at 2500 g. The organic phase was allowed to evaporate for 48 h and then reconstituted in 100% chloroform. Duplicate 500 *μ*L aliquots were dried overnight to determine total lipids. Additional aliquots were used to determine triglyceride content using Infinity^™^ Triglycerides Reagent (Thermo Fisher Scientific, Waltham, MA) and cholesterol levels using an enzymatic kit (Wako Diagnostics, Richmond, VA).

### Serum lipid analysis

Whole blood was collected from the vena cava of overnight‐fasted mice. Serum was separated and total cholesterol, HDL, and LDL were analyzed using enzymatic kits from Wako Diagnostics, triglycerides using Infinity^™^ Triglycerides Reagent and nonesterified free fatty acids using an enzymatic kit from Zenbio (Research Triangle Park, Durham, NC).

### Glucose and insulin tolerance tests

Overnight‐fasted mice were used for glucose tolerance tests (GTTs). At 7 am, mice were anesthetized using isoflurane, their tails snipped, and the mice were allowed to recover for 2 h. Glucose was then administered (1 mg/g ip assuming a 25 g mouse for the 2 weeks time point, and 30 g mouse for the 10 weeks time point), and tail blood glucose was measured at 0, 15, 30, 60, 90, and 120 min using a OneTouch Ultra glucometer. Additional blood was collected at 0, 30, 60, and 90 min for an insulin ELISA (Crystal Chem, Chicago, IL). For insulin tolerance tests (ITTs), mice were fasted for 6 h (morning to afternoon), anesthetized, and insulin administered ip (1.0 U/kg assuming a 25 g mouse for the 1 weeks time point, and a 30 g mouse for the 9 weeks time point); glucose levels were measured as described above.

### Total mRNA isolation from mouse intestine

Intestine was removed from anesthetized overnight‐fasted mice, sectioned into three equal‐length pieces, and the contents were removed. The tissues were placed in Ambion RNAlater and frozen at −20°C until RNA isolation. Total RNA was isolated from these tissues using the RNeasy Midi Kit from Qiagen (Valencia, CA).

### Real‐time quantitative reverse‐transcriptase PCR analyses

The primers used for PCR analysis have been described (Wheeler et al. 2014). Relative gene expression was determined on a Corbett Rotor‐Gene 3000 real‐time cycler (San Francisco, CA) using gene‐specific cDNA standards containing 10–10^7^ copies diluted in yeast total RNA. Samples (*n* = 4–7) were analyzed in duplicate using Stratagene Brilliant III Ultra‐Fast SYBR Green QRT‐PCR Master Mix (Agilent Technologies, Inc., Santa Clara, CA). A sample (50 ng) of total RNA was analyzed per reaction.

### Statistical analysis

Differences between pairs of values were analyzed by Student's *t*‐test and differences between three or more values were analyzed by ANOVA with Tukey's post‐hoc test. Food consumption, fecal production, and fecal lipids were measured per cage, and *n* was the number of cages analyzed. Gene expression data were converted to log_2_ for representation on a heat map.

## Results

### *Ostα*^*−/−*^ mice are not resistant to WD‐induced weight gain

**Figure 1.**
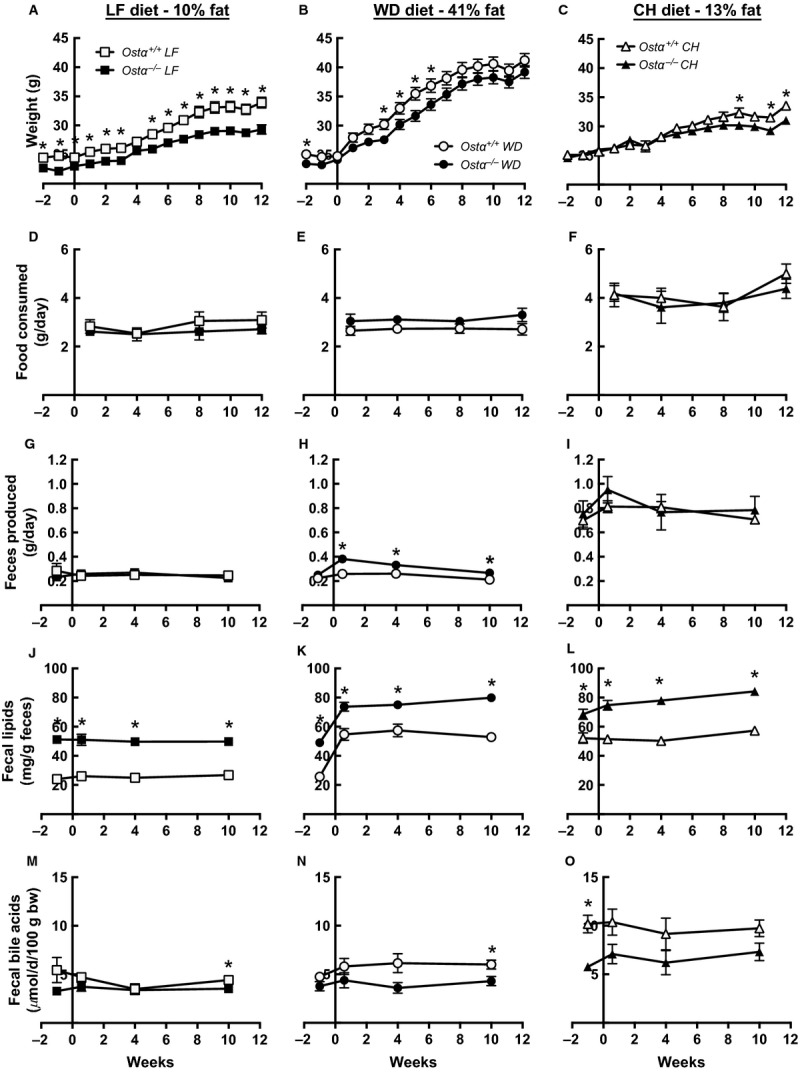
*Ostα*^*−/−*^ mice are not resistant to WD‐induced weight gain yet excrete more lipid than wild‐type mice. Results for wild‐type (open symbols) and *Ostα*^*−/−*^ mice (filled symbols) fed LF (left panels), WD (middle panels), or CH (right panels). Weight gain (A, B, C); food consumption (D, E, F); fecal production (G, H, I); fecal lipid content (J, K, L); and fecal bile acid levels (M, N, O). Values are means ± SE (*n* = 6–14) *P ≤**0.05 versus wild‐type.

Mice were placed on a synthetic low fat control diet (LF) or a synthetic western‐style high‐fat diet (WD) with elevated fat and cholesterol levels (Table 1) to determine whether *Ostα*^*−/−*^ mice were resistant to WD‐induced weight gain. After weaning (~2 months of age), mice were switched from a standard chow diet (CH) to the LF diet for two wk, starting at −2 weeks in figures (Fig. 1). Half of these mice were switched to the WD at wk 0 and half remained on the LF diet for 12 weeks. As an additional control, a group of mice was maintained on standard laboratory chow (CH), which differs substantially from the LF and WD, to allow comparison with defined diets using mice bred and treated identically to those on the defined diets and to confirm previous reports. The group of mice used in the experiments described here was different from those used in previously published studies. On the LF diet, *Ostα*^*−/−*^ mice remained significantly lighter than the wild‐type mice throughout (Fig. 1A). Surprisingly, on the WD both wild‐type and *Ostα*^*−/−*^ mice gained weight rapidly and their weights were not significantly different after 12 weeks (Fig. 1B). Mice gained the least weight on CH and the *Ostα*^*−/−*^ mice on CH weighed significantly less than wild‐type by the end of the study (Fig. 1C).

Food consumption was measured during wks 1, 4, 8, and 12. *Ostα*^*−/−*^ mice tended to eat less than wild‐type mice on the LF diet and more on the WD, although the differences were not statistically significant (Fig. 1D and E). *Ostα*^*−/−*^ mice produced more feces than wild‐type mice on the WD (Fig. 1H) but not on the LF diet (Fig. 1G), consistent with food consumption in the two groups. Mice on the fiber‐rich CH diet ate more, produced more feces and displayed no genotype‐dependent differences in food consumption or fecal production (Fig. 1F and I).

### *Ostα*^*−/−*^ mice excrete more lipid than wild‐type on a WD

The concentration of lipids in feces was much higher in *Ostα*^*−/−*^ than wild‐type mice in all groups (Fig. 1J, K, and L). Total fecal lipid excretion was calculated from the weight of feces and the fecal lipid concentration determined at 10 weeks. Wild‐type and *Ostα*^*−/−*^ mice on LF diets excreted 6.6 and 11.2 mg lipid/day respectively. WD‐fed mice excreted higher amounts (11.2 and 21.3 mg lipid/day for wild‐type and *Ostα*^*−/−*^ mice). CH‐fed mice excreted the most total lipid, 40.5 and 66.0 mg lipid/day (wild‐type and *Ostα*^*−/−*^).

Overall fat absorption was estimated from the amount of food consumed (measured at 12 weeks), percentage of fat in CH, LF, and WD (supplied by manufacturer); and fecal production and lipid content (measured at 10 weeks). On this basis, wild‐type mice absorbed 95, 98, and 82% of fat on LF, WD, and CH respectively. *Ostα*^*−/−*^ mice absorbed lipid less efficiently, with absorption averaging 91, 97, and 67% on LF, WD, and CH diets. Fecal bile acid levels tended to be lower in *Ostα*^*−/−*^ mice on all diets (Fig. 1 M, N, and O).

### *Ostα*^*−/−*^ mice are partially protected against WD‐induced lipid accumulation

**Figure 2.**
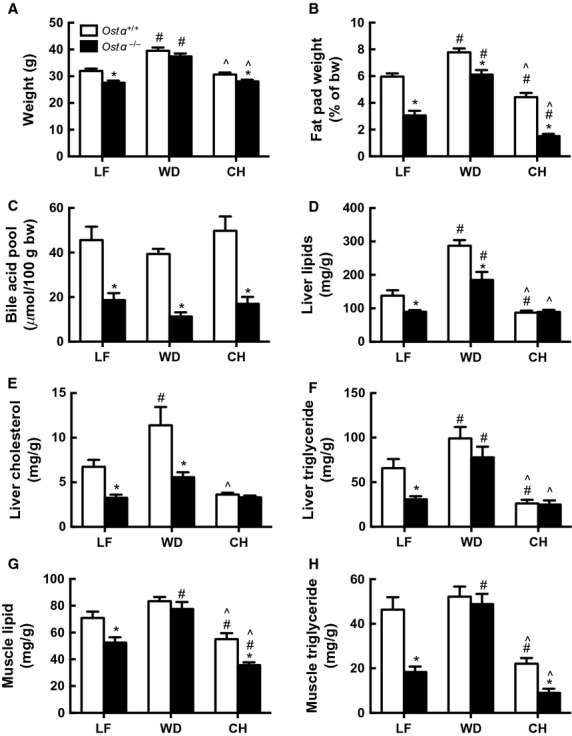
*Ostα*^*−/−*^ mice are partially protected from effects of WD. At 12 weeks, LF‐, WD‐, and CH‐fed mice were fasted overnight and tissues were removed for lipid and bile acid analysis. Total body weights (A); gonadal and perirenal fat pad weights (B); bile acid pool size (C); liver total lipids (D); liver cholesterol (E); liver triglycerides (F); quadricep total lipids (G); and quadricep triglycerides (H). Values are means ± SE (*n* = 6–14). *P ≤**0.05 versus wild‐type; ^#^*P *≤**0.05 versus LF; ^*P *≤**0.05 versus WD.

At the end of the study (12 weeks), the whole body weights of the *Ostα*^*−/−*^ LF‐ and CH‐fed mice were significantly less than those of the wild‐type animals, whereas there were no differences in weight between the two genotypes in the WD‐fed groups (Fig. 2A). The fat pads of *Ostα*^*−/−*^ mice were significantly smaller than those of the wild‐type mice on all three diets (Fig. 2B), although the difference was smallest in mice fed a WD. The fat pads were largest in the WD group and smallest in CH‐fed mice of both genotypes. Bile acid pool levels were reduced by 76%, 65%, and 71% in *Ostα*^*−/−*^ mice on LF, WD, and CH respectively (Fig. 2C). Taken together, the data suggest that the lower bile acids in the lumen of the intestine of *Ostα*^*−/−*^ mice led to greater loss of lipids in feces but did not cause the *Ostα*^*−/−*^ mice to gain less weight on the WD.

In liver, total lipids (Fig. 2D), cholesterol (Fig. 2E), and triglycerides (Fig. 2F) all increased significantly when mice of either genotype were placed on a WD for 12 weeks. Despite this, livers of the *Ostα*^*−/−*^ mice were partially protected from lipid accumulation on both LF and WD. In CH‐fed mice, there were no genotype‐dependent differences in liver lipids, cholesterol, and triglycerides, which were low relative to values on the defined diets.

In muscle, total lipid (Fig. 2G) and triglyceride (Fig. 2H) were lower in *Ostα*^*−/−*^ than wild‐type mice on the LF diet, as seen in liver. In contrast, the protection afforded by the *Ostα*^*−/−*^ geneotype was lost in muscle tissue when animals were placed on a WD; in this case, *Ostα*^*−/−*^ and wild‐type mice had similar lipid levels. Muscle lipid and triglycerides were lower in CH‐fed *Ostα*^*−/−*^ compared with wild‐type mice. The actual levels of muscle lipids and particularly triglycerides were again lower in CH‐fed mice compared to LF‐ of WD‐fed mice.

Both wild‐type and *Ostα*^*−/−*^ mice had significantly increased total serum cholesterol levels on the WD, primarily due to large increases in LDL (Table 2). Total serum cholesterol levels were 17% lower in the *Ostα*^*−/−*^ compared to the wild‐type mice on both the LF and WD. Mice on CH had low total serum cholesterol and barely measurable LDL, and lipid levels in CH‐fed *Ostα*^*−/−*^ mice were no lower than those in wild‐type mice. Triglyceride and free fatty acid concentrations tended to be lower in mice on CH and were not significantly affected by differences in genotype.

**Table 2.** Serum chemistry in wild‐type and *Ostα*^−/−^ mice on LF, WD, and CH diets. Serum was collected from overnight‐fasted mice at 12 weeks. Values are means ± SE (*n *=**12–14). *P ≤**0.05 versus wild‐type; ^#^*P *≤**0.05 versus LF; ^*P *≤**0.05 versus WD

	LF		WD		CH	
	*Ostα* ^+/+^	*Ostα* ^*−/−*^	*Ostα* ^+/+^	*Ostα* ^*−/−*^	*Ostα* ^+/+^	*Ostα* ^*−/−*^
Total cholesterol, mg/dL	141 ± 6	117 ± 4*	230 ± 18 #	191 ± 9 #	69 ± 3 #^	74 ± 4 #^
HDL, mg/dL	60 ± 3	56 ± 2	66 ± 5	71 ± 3 #	43 ± 2 # ^	45 ± 2 # ^
LDL, mg/dL	28 ± 2	22 ± 2	89 ± 16 #	87 ± 19 #	9 ± 1 ^	14 ± 1 ^ *
Triglycerides, mg/dL	45 ± 3	56 ± 6	53 ± 6	45 ± 2	34 ± 2 ^	43 ± 7
Free fatty acids, *μ*mol/L	929 ± 56	853 ± 45	1077 ± 83	939 ± 74	713 ± 61 ^	772 ± 51

### *Ostα*^*−/−*^ mice are marginally protected from WD‐induced insulin resistance

**Figure 3.**
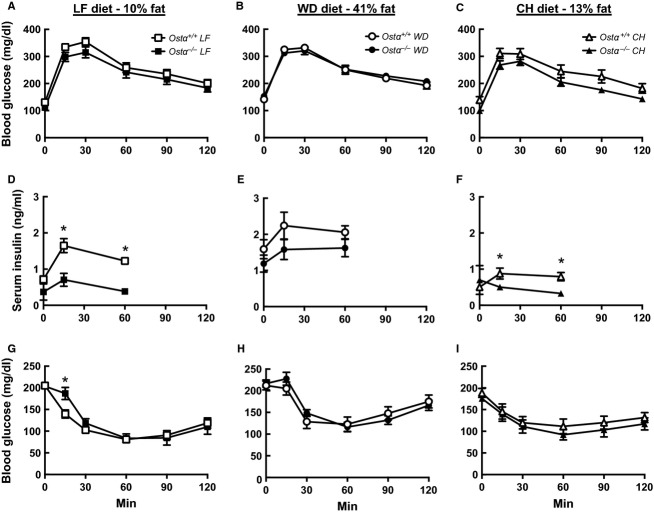
Glucose tolerance and insulin sensitivity. GTTs were performed in LF‐fed (A), WD‐fed (B), and CH‐fed mice at week 10 following an overnight fast. There were no significant differences in the AUC for panels A, B, or C. Body weights were: 31.4 ± 0.9 and 27.1 ± 0.7* g for wild‐type and *Ostα*^*−/−*^ mice on LF diet; 38.7 ± 1.2 and 36.6 ± 1.0 g for wild‐type and *Ostα*^*−/−*^ mice on WD; and 29.6 ± 0.6 and 26.6 ± 0.6* g for wild‐type and *Ostα*^*−/−*^ mice on CH. Additional blood was collected from tail snips at 0, 15, and 60 min for insulin analysis, shown in panels D, E, and F. Insulin tolerance tests were performed in LF‐fed (G), WD‐fed (H), and CH‐fed mice (I) at week 9 following a 6‐h fast. Values are means ± SE (*n* = 12–14) *P ≤**0.05 versus wild‐type.

To test whether *Ostα*^*−/−*^ mice are protected from diet‐induced glucose intolerance and insulin desensitization, glucose tolerance tests (GTTs) and insulin tolerance tests (ITTs) were performed on all groups of mice after 1–2 and 9–10 weeks on the different diets. Fasting glucose concentrations were lower following an overnight fast, which preceded the GTTs, than after the 6 h fast preceding the ITTs. Because results were very similar at the two time points, only the findings at 9–10 weeks are shown (Fig. 3). Fasting blood glucose levels were similar in both genotypes and not significantly altered by diet (Fig. 3A–C and G–I). The changes in blood glucose in response to administration of glucose (GTTs) and insulin (ITTs) were not significantly different for wild‐type and *Ostα*^*−/−*^ mice, although *Ostα*^*−/−*^ mice on LF and CH diets trended toward improved glucose tolerance.

Serum insulin concentrations were measured before and during GTTs (Fig. 3D–F). Fasting insulin levels were higher for both wild‐type and *Ostα*^*−/−*^ mice on the WD compared to the LF and CH diets. These results suggest that mice of both genotypes become less sensitive to insulin on the WD. During the GTTs, insulin levels in the *Ostα*^*−/−*^ mice were significantly lower than those in wild‐type mice on LF and CH diets. In contrast with expectations, the difference in insulin sensitivity was not exaggerated when *Ostα*^*−/−*^ mice were fed a WD; in fact, the difference became smaller. *Ostα*^*−/−*^ mice showed a sharp rise in fasting insulin on a WD and their insulin levels were not significantly lower than those of wild‐type mice during the GTT.

### Gene expression of cholesterol and lipid transporters

Expression of genes involved in cholesterol and lipid transport was analyzed by quantitative RT‐PCR using RNA obtained from the proximal, middle, and lower thirds of the small intestine (Table 3). Differences in the expression of three genes involved in lipid transport, fatty acid translocase (*CD36* or *Fat*), scavenger receptor B‐I (*Srb1*), and Niemann‐Pick C1‐like 1 protein (*Npc1 l1*), were less than 2.5‐fold between genotypes and between the LF and WD. In LF‐fed mice, *CD36, Srb1*, and *Npc1 l1* were either lower or the same in the *Ostα*^*−/−*^ mice compared with wild‐type mice in all segments of the small intestine. In WD‐fed mice, *Ostα*^*−/−*^ and wild‐type mice differed significantly only in the distal segment, where *CD36* and *Srb1* were slightly higher and *Npc1L1* was lower in the *Ostα*^*−/−*^ mice. Wild‐type mice fed WD had slightly decreased or unchanged gene expression levels for *CD36*,* Srb1*, and *Npc1 l1* compared with levels on the LF diet. In contrast, *Ostα*^*−/−*^ mice responded to WD with small increases in the expression of *CD36* and small decreases in expression of *Npc1 l1* throughout the intestine, although most of the differences were not significant.

**Table 3.** Expression of genes important for lipid and cholesterol transport in the intestines of mice fed LF or WD. Columns labeled *Ostα*^+/+^ and *Ostα*^−/−^ show gene expression normalized to β‐actin expression and then multiplied by 100. Values are means ± SE (*n* = 4–7). *P ≤ 0.05 versus wild‐type; ^#^*P* ≤ 0.05 versus LF; ^*P* ≤ 0.05 versus WD. Abca1, ATP‐binding cassette, subfamily A, member 1; *Abcg5*, ATP‐binding cassette, subfamily G, member 5; *Abcg8*, ATP‐binding cassette, subfamily G, member 8; *CD36*, fatty acid translocase (FAT)/*CD36*;* Npc1L1*, Niemann‐Pick disease, type C1‐like 1; *Srb1*, scavenger receptor class B, member 1

Gene	LF		WD		CH	
	*Ostα* ^*+/+*^	*Ostα* ^*−/−*^	*Ostα* ^*+/+*^	*Ostα* ^*−/−*^	*Ostα* ^*+/+*^	*Ostα* ^*−/−*^
Proximal small intestine						
* Abca1*	6.4 ± 0.7	2.0 ± 0.1 *	7.8 ± 0.7	4.0 ± 0.6 *#	1.60 ± 0.09 #^	1.18 ± 0.11 *^
* Abcg5*	2.9 ± 0.3	1.2 ± 0.1 *	2.9 ± 0.1	2.6 ± 0.5 #	2.7 ± 0.3	1.9 ± 0.2
* Abcg8*	3.9 ± 0.4	1.8 ± 0.2 *	4.2 ± 0.4	3.3 ± 0.4 #	1.6 ± 0.2 # ^	1.4 ± 0.2 ^
* CD36*	0.14 ± 0.02	0.09 ± 0.01	0.11 ± 0.01	0.13 ± 0.01	0.42 ± 0.03 #^	0.62 ± 0.09 *#^
* Npc1L1*	2.7 ± 0.2	2.1 ± 0.1	1.6 ± 0.1 #	1.9 ± 0.1	1.88 ± 0.13 #	2.65 ± 0.06 *#^
* Srb1*	0.099 ± 0.015	0.064 ± 0.006 *	0.080 ± 0.007	0.10 ± 0.01	0.36 ± 0.03 #^	0.54 ± 0.09 #^
Middle small intestine						
* Abca1*	1.3 ± 0.1	0.6 ± 0.1 *	2.4 ± 0.1 #	1.2 ± 0.2 *#	1.12 ± 0.07 ^	0.59 ± 0.08 *^
* Abcg5*	0.23 ± 0.02	0.12 ± 0.01 *	0.29 ± 0.04	0.16 ± 0.02 *	1.4 ± 0.1 #^	1.1 ± 0.1 #^
* Abcg8*	2.5 ± 0.3	1.3 ± 0.1 *	2.8 ± 0.2	2.4 ± 01 #	1.08 ± 0.06 # ^	1.17 ± 0.16 ^
* CD36*	0.12 ± 0.01	0.11 ± 0.01	0.15 ± 0.01	0.15 ± 0.01 #	0.078 ± 0.008 # ^	0.104 ± 0.013 *^
* Npc1L1*	2.8 ± 0.1	2.7 ± 0.2	2.3 ± 0.1 #	2.2 ± 0.1 #	1.65 ± 0.08 #^	2.32 ± 0.19 *
* Srb1*	0.15 ± 0.02	0.13 ± 0.02	0.12 ± 0.01	0.10 ± 0.01	0.047 ± 0.004 #^	0.055 ± 0.004 #^
Distal small intestine						
* Abca1*	1.9 ± 0.1	0.9 ± 0.1 *	3.9 ± 0.3 #	1.4 ± 0.1 *	2.2 ± 0.4 ^	1.2 ± 0.2
* Abcg5*	0.61 ± 0.04	0.32 ± 0.03 *	0.84 ± 0.08	0.37 ± 0.04 *	1.7 ± 0.2 #^	1.4 ± 0.2 #^
* Abcg8*	1.9 ± 0.1	0.9 ± 0.1 *	2.5 ± 0.1	1.1 ± 0.1 *	1.9 ± 0.2	1.6 ± 0.3 #
* CD36*	0.054 ± 0.003	0.054 ± 0.003	0.041 ± 0.002 #	0.069 ± 0.006 *#	0.020 ± 0.001 #^	0.029 ± 0.004 #^
* Npc1L1*	2.1 ± 0.1	0.9 ± 0.1 *	1.8 ± 0.1 #	0.8 ± 0.1 *	1.4 ± 0.1 #^	0.9 ± 0.1 *
* Srb1*	0.093 ± 0.007	0.091 ± 0.003	0.070 ± 0.006 #	0.102 ± 0.008 *	0.037 ± 0.003 #^	0.050 ± 0.005 #^

Expression of *Abcg5* and *Abcg8*, cholesterol efflux genes on the apical side of enterocytes, was markedly lower in *Ostα*^*−/−*^ compared with wild‐type mice in all sections of the small intestine on the two defined diets. WD feeding significantly increased *Abcg5* and *Abcg8* in the proximal segment of *Ostα*^*−/−*^ mice. Expression of the basolateral cholesterol transporter *Abca1* was significantly lower in all areas of the *Ostα*^*−/−*^ small intestine compared with wild‐type on all diets. When fed WD, both *Ostα*^*−/−*^ and wild‐type mice increased expression of *Abca1*.

Expression of cholesterol and lipid transport genes differed much more between CH and LF diets than between the defined LF and WD (Table 3). CH‐fed *Ostα*^*−/−*^ mice had higher levels of *Srb1*,* Npc1 l1*, and *CD36* compared to wild‐type throughout the intestine except that *Npc1 l1* was lower in the distal small intestine, as also seen on LF and WD. In comparison with mice fed the LF diet, CH‐fed mice of both genotypes had greater expression of *CD36* and *Srb1* in the proximal small intestine and lower expression elsewhere. There were no genotype‐dependent differences in expression of *Abcg5* by mice on CH, and expression of *Abcg5* was significantly higher in the middle and distal intestines of CH‐fed compared with LF‐fed animals. In contrast, *Abcg8,* the partner of *Abcg5*, was generally lower in CH‐fed mice. *Abca1* was lower in *Ostα*^*−/−*^ relative to wild‐type mice on CH as well as on the defined diets. In the proximal intestine, wild‐type mice expressed much less *Abca1* than they did on LF or WD.

## Discussion

The major finding of this study is that *Ostα*^*−/−*^ mice are not protected from weight gain and many of the lipid changes caused by a western‐style diet. This result was unexpected, because *Ostα*^*−/−*^ mice are resistant to the weight gain, lipid accumulation in liver and muscle, and insulin resistance associated with normal aging (Wheeler et al. 2014). It seemed likely that poor lipid absorption would have an even greater impact when *Ostα*^*−/−*^ mice were fed a WD, which provides 41% of calories from fat compared to 12.7% in the chow diet used in the earlier aging study. After 12 weeks of WD feeding, body weights, fat pad weights, liver and muscle lipids, and serum cholesterol rose in both genotypes and the increases were actually larger in *Ostα*^*−/−*^ mice even though their bile acid pools remained very low. *Ostα*^*−/−*^ mice continued to have lower hepatic lipids and abdominal fat than wild‐type, but muscle lipids and triglycerides had become the same.

*Ostα*^*−/−*^ mice might be expected to respond to a high‐fat diet in the same manner as other genetically altered mice with a decreased bile acid pool. *Gpbar1*^*−/−*^ mice (TGR5) have a ~25% decrease in bile acid pool size but gain weight at the same rate or slightly more rapidly than wild‐type mice on both standard chow and high‐fat diets (Maruyama et al. 2006), indicating that this relatively small change in bile acid does not prevent diet‐induced obesity. *Cyp7a1*^*−/−*^ mice have an ~80% reduction in bile acid pool size and a ~15% decrease in overall lipid absorption (Ishibashi et al. 1996; Schwarz et al. 1996, 1998, 2001). Like *Ostα*^*−/−*^mice, *Cyp7a1*^*−/−*^mice have very low cholesterol absorption (Schwarz et al. 2001; Lan et al. 2012) and exhibit only a marginal increase in liver cholesterol after consuming a diet with elevated cholesterol (Fig. 2 and Schwarz et al. 2001). *Cyp7a1*^*−/−*^ mice die shortly after birth unless their diet is supplemented with cholic acid and vitamins. Interestingly, mice overexpressing hepatic *Cyp7a1* have ~2.5‐fold increase in bile acid pool and are protected from WD‐induced weight gain, apparently due to an increase in energy expenditure (Li et al. 2010).

Animals efficiently increase intestinal fat absorption when presented with high‐fat diets (Lynes et al. 2011; Duca et al. 2013; Buttet et al. 2014). This increase in fat absorption is a complex process that appears to involve a number of mechanisms, including an increase in absorptive surface area and the number of enterocytes in the proximal and middle portions of the small intestine where the majority of fatty acid and cholesterol absorption occurs (Duca et al. 2013). Other mechanisms include micellar expansion, increased lipid transport into enterocytes across the apical membrane, and increased chylomicron and HDL transport into lymph and blood across the basolateral membrane (Buttet et al. 2014). Finally, high‐fat diets lead to changes in the levels of proteins involved in lipid and cholesterol transport (Buttet et al. 2014).

**Figure 4.**
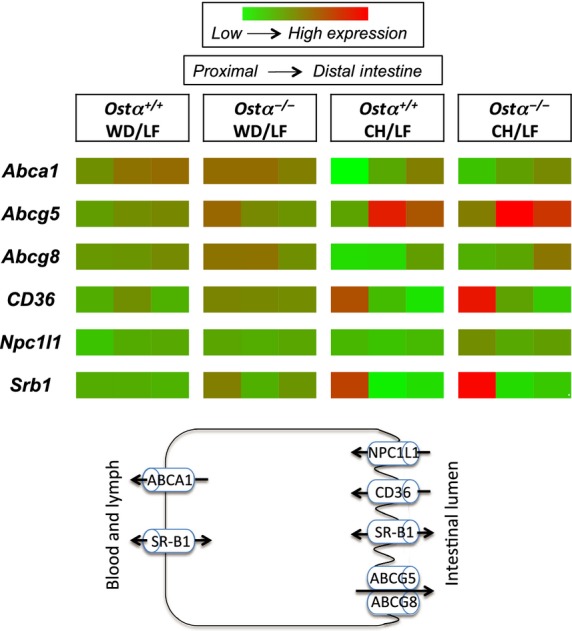
Effect of genotype, diet, and intestinal segment on expression of genes involved in lipid and cholesterol transport. Data from Table 3 are shown schematically in heat map format using the same scale for all data, depicting the three intestinal segments for each genotype and diet. The scheme portrays major functions of the proteins.

Proteins implicated in transport of cholesterol and/or fatty acids into enterocytes at the brush border membrane include fatty acid translocase (CD36) (Abumrad et al. 1993; Drover et al. 2008), scavenger receptor B‐I (SR‐BI) (Hauser et al. 1998; Bietrix et al. 2006) and Niemann‐Pick C1‐like 1 protein (NPC1L1) (Altmann et al. 2004), shown schematically in Fig. 4. In general, expression of these genes decreased slightly when wild‐type mice were switched from a LF to a WD. In *Ostα*^*−/−*^ mice, *CD36* was slightly increased by WD feeding whereas *Srb1* and *Npc1 l1* did not change appreciably. CD36 is increased in various high‐fat diets, especially in the jejunum (Petit et al. 2007; Lynes et al. 2011; de Wit et al. 2011; Desmarchelier et al. 2012). Uptake of long‐chain fatty acids via CD36 is also importantly regulated by phosphorylation and subcellular localization (Lynes et al. 2011) and deletion of *CD36* does not inhibit intestinal lipid uptake (Tran et al. 2011). In addition to facilitating transport of lipids and cholesterol, CD36 may act as a lipid sensor that increases lipid absorption through cell signaling (Abumrad and Davidson 2012; Buttet et al. 2014). SR‐BI is located on both the apical and basolateral membranes of enterocytes, and appears to play a role in cholesterol and lipid uptake from the intestinal lumen as well as cholesterol efflux into HDL particles (Hauser et al. 1998). *Npc1 l1*, which transports cholesterol and phytosterols into enterocytes (Davis et al. 2004), decreased in wild‐type mice with WD feeding. Decreased expression of *Npc1 l1* on high‐cholesterol diets has been reported before and may reflect negative feedback (Abumrad and Davidson 2012; Desmarchelier et al. 2012; Buttet et al. 2014). *Ostα*^*−/−*^ mice clearly adapt to a WD by increasing lipid absorption, and the gene expression data suggest that the mechanisms involved may include increased intestinal expression of CD36 but not of NPC1L1.

Proteins encoded by *Abcg5* and *Abcg8* form a heteromeric complex that functions as a cholesterol and phytosterol efflux transporter on the brush border membrane of enterocytes (Yu et al. 2002; Graf et al. 2003). Effluxed cholesterol can then be taken up by enterocytes further down in the intestine or eliminated in feces. *Abcg5* and *Abcg8* were considerably lower in *Ostα*^*−/−*^ than wild‐type mice on LF diets but higher for *Ostα*^*−/−*^ mice on western‐style relative to CH diets. These findings are consistent with reports that high‐cholesterol diets increase expression of *Abcg5* and *Abcg8* (Berge et al. 2000; Repa et al. 2002), whereas cholesterol‐free high‐fat diets decrease expression (de Vogel‐ den Bosch et al. 2008; de Wit et al. 2011).

ABCA1 is present on the basolateral side of the enterocyte and is important for cholesterol absorption and the formation of HDL from chylomicrons (Brunham et al. 2006). *Abca1* expression was higher in wild‐type than *Ostα*^*−/−*^ mice in all areas of the small intestine on all diets. After a switch to the WD, expression of *Abca1* increased markedly, particularly in *Ostα*^*−/−*^ mice. This increase likely reflects the increase in dietary cholesterol because *Abca1* is low on cholesterol‐free high‐fat diets (de Vogel‐ den Bosch et al. 2008; de Wit et al. 2011).

A direct comparison of the effect of the three different diets on gene expression is shown as the heat map in Fig. 4. It is readily apparent that differences between CH and LF diets were much greater than differences between LF and WD. *CD36* and *Srb1* in the proximal intestine and *Abcg5* in the middle section (jejunum) were expressed at the highest levels in CH‐fed mice of both genotypes. The high expression of *CD36* and *Srb1* is not easily reconciled with lower lipid absorption observed for animals on CH. All of the gene expression data should be interpreted cautiously, however. Message levels were analyzed in tissues from overnight‐fasted animals. This protocol may have missed changes in expression of lipid transporter genes that occurred shortly after eating. In addition, changes in the synthesis, activity, and intracellular localization of lipid‐binding and transport proteins may not correspond to changes in message levels. The lack of changes in mRNA encoding intestinal lipid transporters also suggests the involvement of liver in adaptation to a high‐fat diet. It is likely that responses initiated by bile acid signaling through FXR and TGR5 contribute to the different effects of dietary changes on wild‐type and *Ostα*^*−/−*^ mice.

Muscle from aged *Ostα*^*−/−*^ mice displayed lower triglyceride content and stronger insulin responses than muscle from wild‐type mice; we proposed that these differences contribute to improved glucose tolerance in the *Ostα*^*−/−*^ mice (Wheeler et al. 2014). The data obtained in the present diet study support this mechanism. After 12 wk on the WD, *Ostα*^*−/−*^ and wild‐type mice had similar muscle lipid and triglyceride levels and *Ostα*^*−/−*^ mice no longer fared better in GTTs and ITTs. Predictably, insulin levels were higher on WD than on LF or CH. *Ostα*^*−/−*^ mice were able to maintain the same blood glucose as wild‐type with lower serum insulin levels even on the WD, perhaps due to lower liver lipids and abdominal fat tissue.

The results reported here strengthen the argument for choosing the appropriate control diet in investigations of diet‐induced obesity, glucose homeostasis, and lipid metabolism (Thigpen et al. 2004; Warden and Fisler 2008; Benoit et al. 2013). Although the LF diet was an appropriate control for the WD, characterizing the differences between CH and the defined diets was also valuable because almost all previous studies used animals on CH. The defined WD and LF diets contained casein as the major source of protein, cellulose as the source of fiber, and sucrose as a major source of carbohydrates; high corn starch provided additional carbohydrate in the LF diet. CH contained protein from a variety of vegetable sources, high fiber, and mostly complex carbohydrates; CH also contained phytoestrogens from soy. High sucrose in the defined diets is metabolized to glucose and fructose, and fructose consumption is linked to increases in serum triglycerides, hepatic triglycerides, and muscle lipids (Stanhope and Havel 2008). High fructose may have contributed to higher liver and muscle lipids on LF‐ compared to CH‐fed diets. Another factor may have been the high dietary fiber in CH, which protects against increases in serum and hepatic cholesterol and weight gain with a high‐fat, high‐cholesterol diet (van Bennekum et al. 2005). Some of the differences between CH and LF diets may also have been due to phytosterols, which can lower serum cholesterol by reducing intestinal cholesterol absorption (Normen et al. 2000; Brufau et al. 2008).

Initial characterizations of the *Ostα*^*−/−*^ mouse described favorable changes in lipid metabolism, body composition, age‐related weight gain, and longevity. The experiments reported here show that *Ostα*^*−/−*^ mice are not protected from many of the adverse consequences of a western‐style diet. In fact, it seems unlikely that *Ostα*^*−/−*^ mice would continue to have lower abdominal fat stores or lower hepatic lipids with prolonged western diet feeding. To the extent that data obtained with genetically altered mice can be extrapolated to humans, the present results suggest that OST is unlikely to be a useful drug target for the “metabolic syndrome” characterized by central obesity, hypertriglyceridemia and low HDL, hyperglycemia, and hypertension. In response to high dietary fat, *Ostα*^*−/−*^ mice increased lipid absorption without increasing bile acid production and without major changes in the expression of a number genes involved in lipid transport. Future studies may identify additional mechanisms for this adaptation.

## Conflict of Interest

No conflicts of interest, financial or otherwise, are declared by the authors.
